# Synthesis, Characterization, and Adsorption Properties of Nitrogen-Doped Nanoporous Biochar: Efficient Removal of Reactive Orange 16 Dye and Colorful Effluents

**DOI:** 10.3390/nano13142045

**Published:** 2023-07-11

**Authors:** Simon Ekman, Glaydson Simoes dos Reis, Ewen Laisné, Julie Thivet, Alejandro Grimm, Eder Claudio Lima, Mu. Naushad, Guilherme Luiz Dotto

**Affiliations:** 1 Umeå University, SE-901 83 Umeå, Sweden; simonoscekman@gmail.com; 2 Department of Forest Biomaterials and Technology, Biomass Technology Centre, Swedish University of Agricultural Sciences, SE-901 83 Umeå, Sweden; ewen.laisne@mines-albi.fr (E.L.); julie.thivet@enscm.fr (J.T.); alejandro.grimm@slu.se (A.G.); 3 IMT Mines Albi-Carmaux, 81000 Albi, France; 4 Ecole Nationale Supérieure de Chimie de Montpellier, 34090 Montpellier, France; 5 Federal University of Rio Grand do Sul (UFRGS), Porto Alegre 90010-150, RS, Brazil; profederlima@gmail.com; 6 Department of Chemistry, College of Science, King Saud University, P.O. Box 2455, Riyadh 11451, Saudi Arabia; shad123@gmail.com; 7 Research Group on Adsorptive and Catalytic Process Engineering (ENGEPAC), Federal University of Santa Maria, Av. Roraima, 1000-7, Santa Maria 97105-900, RS, Brazil; guilherme_dotto@yahoo.com.br

**Keywords:** spruce bark waste, nitrogen-doped biochar, adsorption, dyes, synthetic effluents

## Abstract

In this work, nitrogen-doped porous biochars were synthesized from spruce bark waste using a facile single-step synthesis process, with H_3_PO_4_ as the chemical activator. The effect of nitrogen doping on the carbon material’s physicochemical properties and adsorption ability to adsorb the Reactive Orange 16 dye and treat synthetic effluents containing dyes were evaluated. N doping did not cause an important impact on the specific surface area values, but it did cause an increase in the microporosity (from 19% to 54% of micropores). The effect of the pH showed that the RO-16 reached its highest removal level in acidic conditions. The kinetic and equilibrium data were best fitted by the Elovich and Redlich–Peterson models, respectively. The adsorption capacities of the non-doped and doped carbon materials were 100.6 and 173.9 mg g^−1^, respectively. Since the biochars are highly porous, pore filling was the main adsorption mechanism, but other mechanisms such as electrostatic, hydrogen bond, Lewis acid-base, and π-π between mechanisms were also involved in the removal of RO-16 using SB-N-Biochar. The adsorbent biochar materials were used to treat synthetic wastewater containing dyes and other compounds and removal efficiencies of up to 66% were obtained. The regeneration tests have demonstrated that the nitrogen-doped biochar could be recycled and reused easily, maintaining very good adsorption performance even after five cycles. This work has demonstrated that N-doped biochar is easy to prepare and can be employed as an efficient adsorbent for dye removal, helping to open up new solutions for developing sustainable and effective adsorption processes to tackle water contamination.

## 1. Introduction

Water safety is one of the most important issues worldwide due to the rapid increases in population and industrial activities [1]. Researchers have been focusing on the control and removal of organic pollutants from water bodies and wastewaters, including phenols [2], dyes [3], and pharmaceuticals [4], among others. Dyes are classified as one of the highest-priority contaminants due to their non-biodegradable nature. When discharged in water bodies, dyes hamper photosynthesis, causing damage to aquatic ecosystems, as well as posing risks to living organisms. Therefore, is imperative to treat colorful effluents before they become a threat to the environment.

Several methods have been proposed for the treatment of colorful effluents, including biological processes, advanced oxidation processes, filtration processes, photocatalytic methods, and adsorption [5,6,7,8,9,10]. However, among these methods, adsorption is considered the easiest operation; it is less costly and more environmentally friendly because it does not use chemical reagents and does not generate by-products [11,12,13].

To design an effective water treatment process, the selection of the adsorbent is a crucial step. Activated carbon (ACs)/biochars are the most widely used adsorbents worldwide due to their intrinsic properties of high surface area and surface functionalities [14]. Biochars are successfully produced from biomass wastes such as coconut shells [15], sludges [16], wood wastes [17], and other by-products. In this work, spruce bark waste was utilized to produce carbon adsorbents. Spruce bark is a material that is commonly rejected by industries and is currently used as fuel in heat and power plants, but this approach is no longer a sustainable solution. A good alternative usage of this bark is as a precursor for producing ACs/biochars, which have an outstanding ability to absorb various contaminants.

Good adsorption ability is also associated with the material’s surface properties, due to the presence of surface functionalities. To boost the surface activity of any carbon material, heteroatom doping has been proven to be a successful strategy [18,19,20]. Heteroatom doping strategies using cheap and abundant elements such as nitrogen (N) have become very popular because they do indeed boost the product’s adsorptive performance in adsorbing organic pollutants, including dyes.

Lian et al. [21] used crop straws as a carbon precursor to produce nitrogen-doped biochars using NH_3_ as a dopant. The authors found that the incorporation of N into the carbon matrix led to a more microporous structure, and 8.8% of N was incorporated into the carbon matrix. When employed in the removal of acid orange 7 and methyl blue dyes, the doped sample exhibited a much better removal rate than the non-doped sample (~15 times higher). Such a performance was explained by the incorporation of nitrogen in the biochar structure causing changes in its electron density distribution, besides creating new functional groups that boosted the adsorption of the two dyes due to the electrostatic interactions between the doped surface and the dyes.

Herein, we aim to produce and characterize efficient N-doped activated biochar, produced using spruce bark waste, and apply it in the removal of Reactive Orange 16 dye (RO-16). The spruce bark doping was carried out using melamine (C_3_H_6_N_6_) as the doping agent and phosphoric acid (H_3_PO_4_) was used as the activation agent; it was then pyrolyzed at 900 °C. The effect of the doping process was successfully evaluated by employing the doped and non-doped carbons in the removal of RO-16. In addition, the effectiveness of the adsorbents in removing contaminants from synthetic effluents containing different dyes and other contaminants, to simulate real effluents, was also tested.

## 2. Materials and Methods

### 2.1. Materials Preparation

Before biochar preparation, spruce bark waste was ground and sieved at a particle size of between 1 and 3 mm. The doping/activation was carried out in a one-step process. First, the spruce bark was combined with the activation agent (phosphoric acid at 50%) at a ratio of 1:4 (precursor: H_3_PO_4_, wt %:wt %). Then, taking into account the weight of the carbon precursor, a ratio of 0.5:1 (melamine:spruce bark) was implemented. The mixture was mixed by hand for 5 min until it formed a homogenous paste and was then dried in an oven at 60 °C for 24 h. The pyrolysis step was carried out in a stainless-steel reactor placed inside an electrical furnace (Carbolite Gero ELF 11/14) at 900 °C, under an inert atmosphere (constant nitrogen flow) with a heating rate of 10 °C/min. Once the inside of the reactor reached the target temperature, it was maintained for 60 min. When the reaction was completed, the oven was turned off and the pyrolysed carbons were allowed to cool to room temperature. The carbons were washed with hot water until a neutral pH was reached and were then dried at 105 °C overnight. The carbon samples were labeled as SB-Biochar (non-doped) and SB-N-Biochar (nitrogen-doped biochar).

### 2.2. Materials Characterization

In order to determine the textural characterization of the samples, a Tristar 3000 device was used. To calculate the SSA (specific surface area), the BET principle was used. The pore characteristics were obtained, based on the BJH principle. Analysis of the carbon materials’ surface morphology was conducted using a scanning electron microscopy (SEM) method via FESEM (Zeiss Merlin, Oberkochen, Germany) equipment. The degree of graphitization and level of disorder of the carbon structure of SB-Biochar and SB-N-Biochar was determined using Raman spectroscopy. The spectra were obtained using a Bruker BRAVO spectrometer. Analysis of the surface functionalities was carried out via X-ray photoelectron spectroscopy (XPS). The value for the point of zero charge (pH_pzc_) was obtained by plotting the measured zeta potential value, with the pH measured via a Zetasizer Nano ZSE 3700 (Malvern Instrument Co., Malvern, UK) at 298 K, identifying a pH varying from 2 to 10.

### 2.3. Adsorption Experiments

The adsorption experiments were made using a stock solution of Reactive Orange 16 at 1200 ppm, which was diluted into multiple concentrations (30–1200 ppm) [22,23,24,25,26]. The formula of RO-16 is shown in Supplementary Figure S1 (see Supplementary Materials). The absorbent dosage was set up as 30 mg in 20 mL (1.5 g L^−1^) and placed into Falcon tubes of 50 mL. The tubes were then placed in a shaker (IKA KS250) for up to 6 h. After shaking, the tubes were centrifuged to separate the suspended carbon from the RO-16 solution [27,28,29,30,31,32]. In order to measure the remaining RO-16 concentration, the solutions were subjected to measurement via UV spectrophotometry, using a Shimadzu UV-1800 operated at a λ_max_ level of 494 nm. Further explanation is given in the Supplementary Materials.

### 2.4. Kinetic, Equilibrium Models, Regeneration, and Synthetic Effluents

The kinetic data were evaluated using the pseudo-first-order (PFO), pseudo-second-order (PSO), and Elovich models. Isotherms were fitted using the Freundlich, Langmuir, and Redlich–Peterson models. The relevant equations are given in the Supplementary Materials.

For the regeneration of the biochars, SB-Biochar and SB-N-Biochar were first saturated with RO-16 and washed with water to remove any unadsorbed dye. Then, the saturated samples were dried overnight in an oven at 70 °C. The saturated and dried biochars were put in contact with an eluent composed of 0.25 M NaOH +20% EtOH and were then agitated for 4 h to promote the liberation of the adsorbed RO-16 molecules. The desorbed RO-16 was separated from the biochar by centrifugation. Again, the biochars were washed with water to remove the eluent and dried overnight in an oven at 70 °C. This procedure was repeated until five cycles were completed. The adsorption capacity of the reused biochar was measured at each cycle.

The synthetic effluents were prepared by mixing several dyes with other organic compounds and some inorganic salts, which are commonly present in real industrial effluents [14,18]. Two different effluents were made, with different dyes and concentrations, which are presented as the effluents shown in the Supplementary Materials, in Table 1. The aim of using synthetic effluents is to test the performance of adsorbents in the treatment of a mixture of dyes and compounds similar to industrial effluents released in the dyeing process.

## 3. Results and Discussion

### 3.1. Physicochemical Characterization of the Produced Biochars

**Figure 1:**
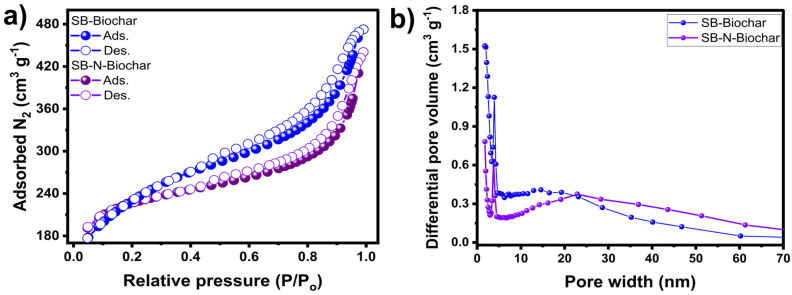
The N_2_ adsorption-desorption curves (**a**) and pore size distribution (**b**) for the SB-Biochar and SB-N-Biochar.

The physical features, including BET surface area, micropore and mesopore structures, and pore volume, are essential for researchers to better correlate and understand the material’s efficiency when applied as an adsorbent in an adsorption process. The N_2_ isotherm was employed to study the SB-Biochar and SB-N-Biochar porosity features (see Figure 1). According to IUPAC, the curves can be classified as type IV due to the hysteresis loop, which is indicative of mesoporous materials.

However, the presence of microporous features is also observed in both materials. The pore size distribution that is highlighted in Figure 1b corroborates the fact that micro- and mesopores are present in both samples. A fraction of pores with diameters of up to 2 nm (micropores) and a larger fraction of less than 5 nm (small mesopores) were observed. Non-doped samples exhibited fractions with larger mesopores compared to those of the doped materials. It seems that the melamine doping created smaller mesopores. Such differences in pore size distribution in the prepared carbons could lead to better adsorptive properties. To further evaluate the porosity features of the materials, the BET surface areas were accessed (Table 1). The results show that the SSA of both biochars was high; however, the melamine doping provoked a small reduction in the BET surface areas for both temperatures. The same trend was observed in other works dealing with the N-doping of similar precursors [22]. The surface areas obtained by the authors were 1086 m^2^ g^−1^ (for non-doped biochars) and 1003 m^2^ g^−1^ (for nitrogen-doped biochars).

**Figure 2:**
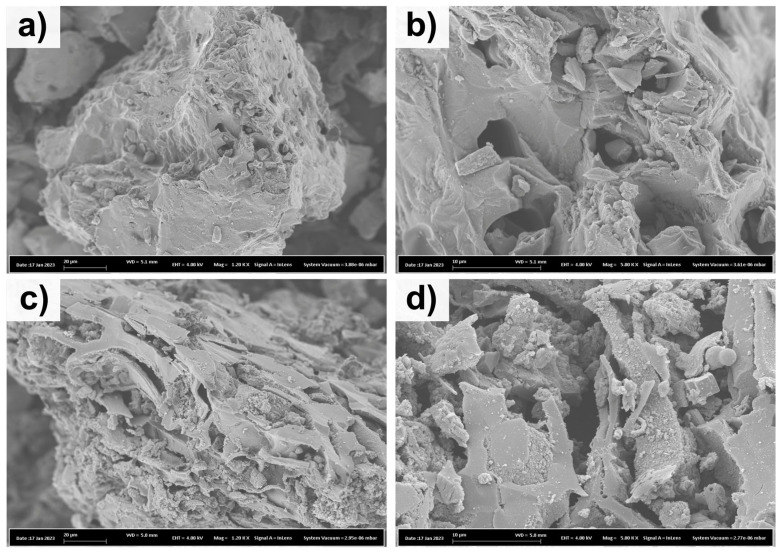
Scanning electron microscopy images of SB-Biochar at 1.2 Kx (**a**) and 5.0 Kx (**b**), with SB-N-Biochar images at 1.2 Kx (**c**) and 5.0 Kx (**d**).

The effect of nitrogen doping on the morphology features of SB-Biochar and SB-N-Biochar was investigated via FESEM (Figure 2). It seems that the doping process provoked significant changes in the carbon morphologies. The SB-N-Biochar seems to have a significantly rougher surface with more cavities and a less intact structure, which could be attributed to the N-doping effect creating more physical defects. It is reported that carbonaceous materials rich in defects are suitable for employment as adsorbents because the defects can act as adsorptive sites and boost the materials’ adsorptive properties [18,19].

**Figure 3:**
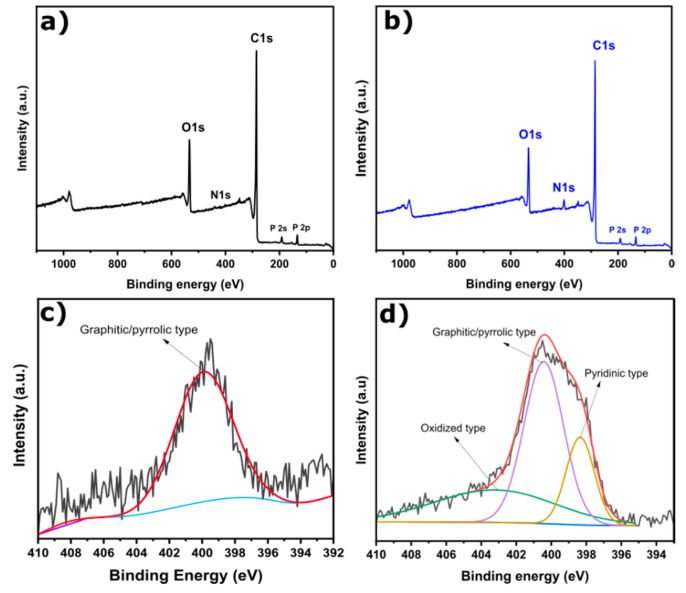
X-ray photoelectron spectroscopy (XPS) analysis of the SB-Biochar and SB-N-Biochar: (**a**) Survey spectrum of SB-Biochar; (**b**) survey spectrum of SB-N-Biochar; (**c**) deconvoluted N 1 s peaks for SB-Biochar; (**d**) deconvoluted N 1 s peaks for SB-N-Biochar.

X-ray photoelectron spectroscopy (XPS) analysis was employed to obtain the chemical composition of the carbon materials regarding their chemical elements and their states. The XPS survey spectra of SB-Biochar and SB-N-Biochar are displayed in Figure 3a and Figure 3b, respectively. Carbon, nitrogen, oxygen, and phosphorus were found in both materials, but the N 1s peak is much more prominent in the SB-N-Biochar, confirming the introduction of N atoms to the biochar structure.

Figure 3c,d show the high-resolution deconvoluted N 1 s spectrum for SB-Biochar and SB-N-Biochar, respectively. Only one peak at 399.6 eV was observed in SB-Biochar, corresponding to graphitic/pyrrolic-N. However, SB-N-Biochar (see Figure 3d) exhibits three separate peaks at 398.3 eV, 400.4 eV, and 403.5 eV, corresponding to pyridinic-N (N1), graphitic/pyrrolic-N (N2), and oxidized-N (N3), respectively [23]. XPS analysis has shown that nitrogen atoms were successfully incorporated into the carbonaceous matrix. Moreover, phosphorus was detected in both materials, which is due to its activation with H_3_PO_4_.

Through XPS analysis, it was possible to obtain a quantitative analysis of each element (see Table 2). The nitrogen content in SB-Biochar was only 0.2%, while that in SB-N-Biochar was 4.1%, which confirms that the doping process incorporated large amounts of N into the biochar structure. Nitrogen doping produced an adsorbent (SB-N-Biochar) with higher amounts of N, indicating that its surface contains larger amounts of functional groups, which directly correlate with the better adsorptive properties gained through electrostatic interactions between these functionalities and the adsorbates.

**Figure 4:**
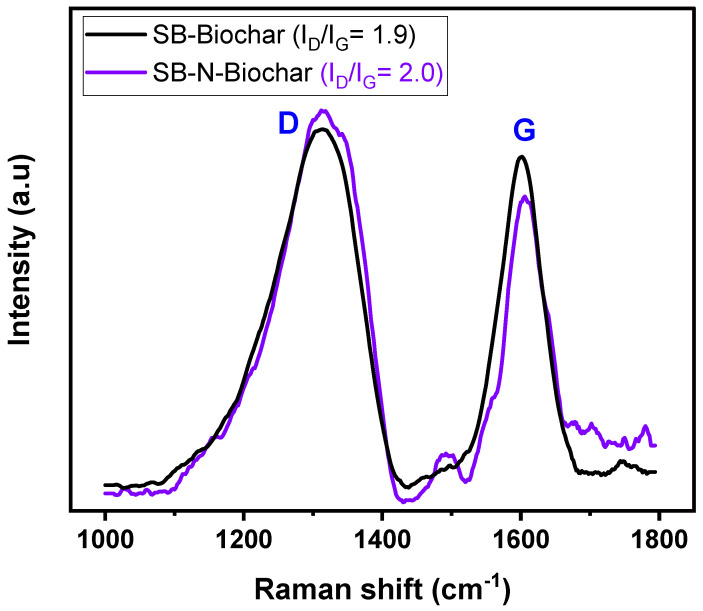
Raman spectra of SB-Biochar and SB-N-Biochar.

Raman analysis is a widely employed technique to evaluate the chemical structure and carbon atom arrangement configuration of carbon-based materials regarding defects and excess charge (doping). The Raman spectra of SB-Biochar and SB-N-Biochar are displayed in Figure 4. The common peaks at 1315 and 1600 cm^−1^ are related to D (defects) and G (sp^2^ hybridized carbon atoms in the form of graphite) peaks, respectively [18,19]. From these peaks, I_D_/I_G_ can be calculated to yield useful information on the degree of graphitization and the level of disorder in the carbon arrangement structure [18,19]. As can be seen, SB-N-Biochar has a slightly higher I_D_/I_G_, indicating a structure that has slightly more defects than that of non-doped biochar. It is reported that the introduction of heteroatoms in the carbon framework leads to more defects. This may be responsible for boosting the doped material’s adsorptive features because defects can act as active adsorption sites [18,19].

### 3.2. Adsorption Studies

#### 3.2.1. The Effect of pH on DCF and RO-16 Adsorption

**Figure 5:**
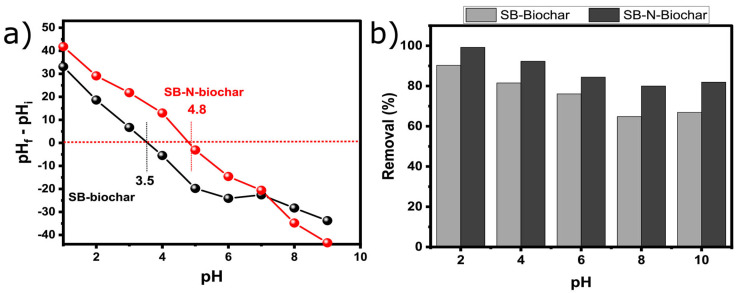
(**a**) Zeta potential of SB-Biochar and SB-N-Biochar; (**b**) the effect of pH on the removal of RO-16.

At the solid–liquid interface, the pH of the system plays a crucial role in the adsorption process [18]. pH_pzc_ acts as the point, whereas the adsorbent’s surface charge is null; this also indicates that for values under pH_pzc_, the biochar’s surface charge is positive, and above that, it is negative. Figure 5a presents the zeta potential curves for SB-Biochar and SB-N-Biochar. The pH_pzc_ values were 3.5 and 4.8 for SB-Biochar and SB-N-Biochar, respectively. It is clear that N-doping led to a decrease in the positive charge of the biochar. Previous studies in the literature show that a pH < pH_pzc_ is favorable for anionic dye adsorption (e.g., RO-16). This means that the SB-N-Biochar surface is positively charged at a pH below 4.8, which favors the removal of anionic RO-16.

The study of the effect of pH on an adsorption system is important because it affects not only the adsorbent surface charge but also the ionization degree of the adsorbate; this has a huge impact on the adsorption performance. The effect of pH on RO-16 removal with SB-Biochar and SB-N-Biochar varied from 4.0 to 10.0 (see Figure 5).

It can be seen that the removal of RO-16 was higher under acidic conditions and it decreased when the pH increased, due to the protonation of amino groups on the surface of SB-N-Biochar; this can be enhanced in an acidic solution, which boosts the adsorption performance between the adsorbents and RO-16 through electrostatic interactions. In addition, in a liquid solution, the Reactive Orange 16 dye molecule dissolves and is transformed into the corresponding dye ions, DSO_3_^−^ and Na^+^ [23]. The surface of SB-N-Biochar is positively charged when the pH of the solution is below 4.8; therefore, it adsorbs RO-16. It has also been widely reported that the pH influences the chemical speciation of RO-16, and different molecule structures can be obtained according to their pKa values. Since the RO-16’s structure has hydroxyl groups, these groups form hydrogen bonds with the adsorbent’s OH sites. The pKa value of RO-16 is 3.75; the dye ionizes into its anion form at a lower pH and affects the electrostatic interaction with the positively charged adsorbent.

#### 3.2.2. Kinetic Study

**Figure 6:**
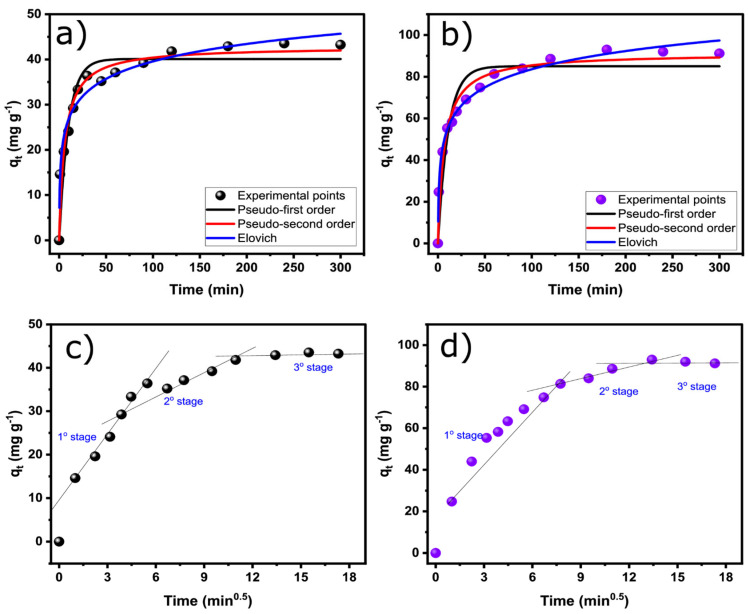
Kinetic curves of RO-16 adsorption onto biochars. SB-Biochar (**a**,**c**); SB-N-biochar (**b**,**d**).

In the adsorption process, the kinetic factor is key to fully understanding the adsorption behavior of any system involving adsorbate–adsorbent. It provides useful information about the adsorbent’s applicability and effectiveness, resulting in a more effective adsorption process [24,25,26]. Figure 6 and Table 3 show the kinetic curves and parameters for RO-16 adsorption on SB-Biochar and SB-N-Biochar adsorbents. The curves show a very similar trend, but SB-Biochar seemed to have reached equilibrium more rapidly, compared to SB-N-Biochar; however, SB-N-Biochar adsorbed twice the amount (91.2 mg g^−1^) compared to SB-Biochar (40 mg g^−1^) at 300 min. This shows that the doping process generates much more active adsorption sites, which boosted the SB-N-Biochar’s adsorptive ability to bind the RO-16 molecules.

From Figure 6, it is clear that the dye molecule is rapidly adsorbed in the first minutes for both adsorbents; this gradually seems to reach equilibrium within 60 and 120 min for SB-Biochar and SB-N-Biochar, respectively. The slightly slower process for SB-N-Biochar could occur because it has more adsorption sites; therefore, it needs more time to become fully saturated.

The kinetic study was accessed by evaluating the suitability of the pseudo-first-order, pseudo-second-order, and Elovich models (Figure 6 and Table 3). The models’ suitability was examined by taking into account the R²_adj_ and SD values [27,28,29,30,31,32] (see the Supplementary Materials). The model that indicates the best suitability for the experimental data must exhibit the highest R²_adj_ and the lowest SD values [27,28,29,30,31,32], which statistically suggest much closer values between the experimental points and the theoretical q-value obtained from the models [27,28,29,30,31,32]. Thus, it is possible to affirm that the Elovich model showed the best suitability for evaluating the kinetic adsorption data of RO-16 on both SB-Biochar and SB-N-Biochar.

To further evaluate the kinetic process and the mass transfer of RO-16, the intra-particle was evaluated (see Figure 6c,d). As seen in Figure 6, the system has three linear stages. The first stage represents the rapid surface adsorption of RO-16 molecules, which occurred in the outermost sites of the biochar adsorbents. The huge initial free adsorption sites of the adsorbent and the massive dissolved solute concentration on the adsorbent surface contribute to the increased mass transfer rate of RO-16 from the bulk solution to the adsorbent surface. Because the adsorbents are very porous, this first stage takes longer. In addition, SB-N-Biochar displayed a first stage that was longer than that of the SB-Biochar; this can be explained by the fact that the doped biochar has more adsorption active sites that needs more time to be saturated, which results in a higher resistance to liquid permeation. Following the first stage, the second stage can be attributed to the diffusion of RO-16 toward the smaller inner pores. This occurs because, as the adsorption persists, the bare binding sites in the adsorbent are gradually occupied by the molecular substance of the solute, establishing an interface between the solute and the solution. Subsequently, slower adsorption of the remaining solute may occur, and the third stage is reached until all adsorption sites are occupied and a state of equilibrium is reached [30,31,32].

#### 3.2.3. RO-16 Adsorption Isotherms

Adsorption isotherms can yield crucial insights into the adsorption mechanism due to the interaction/affinity between adsorbate/adsorbent systems at a constant temperature. Moreover, the isotherm of adsorption can be used to calculate the q_max_ of the adsorbent, a crucial parameter when designing an efficient adsorption process. Many isotherm models are employed in the literature to describe adsorption processes; in this work, the Langmuir, Freundlich, and Redlich–Peterson models were employed to describe the adsorption of RO-16 on biochar adsorbents.

**Figure 7:**
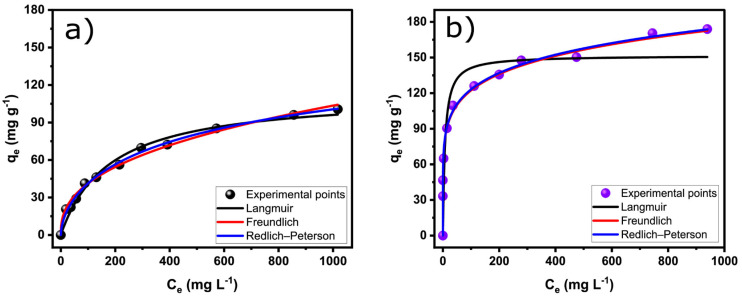
RO-16 equilibrium curves for SB-Biochar (**a**) and SB-N-Biochar (**b**).

The equilibrium results are shown in Figure 7 and Table 4. The curves show that compared to SB-Biochar, the amount of RO-16 adsorbed by SB-N-Biochar sharply increased as the initial concentration was enhanced. As discussed earlier, SB-N-Biochar possesses a greater number of functional groups that serve as active adsorption sites to bind the RO-16 molecules; therefore, it adsorbs much more RO-16 than SB-Biochar at the same initial concentration.

The adsorption suitability data provided by the equilibrium models were evaluated in the same way as in the kinetic evaluation. Based on that evaluation, the Redlich–Peterson model gave the best results. The Redlich–Peterson model combines elements of the Langmuir and Freundlich models; it suggests a hybrid adsorption mechanism that does not follow ideal monolayer adsorption. Evaluating the experimental adsorption capacities of “q” (see the experimental points in the isotherms in Figure 7), the results showed that SB-N-Biochar achieved a q-value of 173.9 mg g^−1^ and SB-Biochar, 100.6 mg g^−1^, proving that N-doping was an effective strategy to boost the biochar’s adsorptive performance.

#### 3.2.4. Comparison with the Literature: Adsorption Capacity

One interesting way of establishing the adsorbent efficiency in terms of its adsorptive performance is to compare the main findings regarding its maximum adsorption capacity with other studies in the literature. Table 5 presents the data for RO-16 adsorption between several adsorbents found in the literature. The table shows that the adsorption capacity of SB-N-Biochar yields among the four highest values in terms of performance, compared to the other adsorbents. However, the highest capacity was achieved by a composite prepared with chitosan, tripolyphosphate, and TiO_2_ (618.7 mg g^−1^); however, its synthesis is much more complex and extremely costly when compared to that of SB-N-Biochar. Thus, compared to other types of adsorbents, the biochars developed in this work can be considered effective cheap adsorbents to treat dye-polluted water.

#### 3.2.5. Mechanism of Adsorption

**Figure 8:**
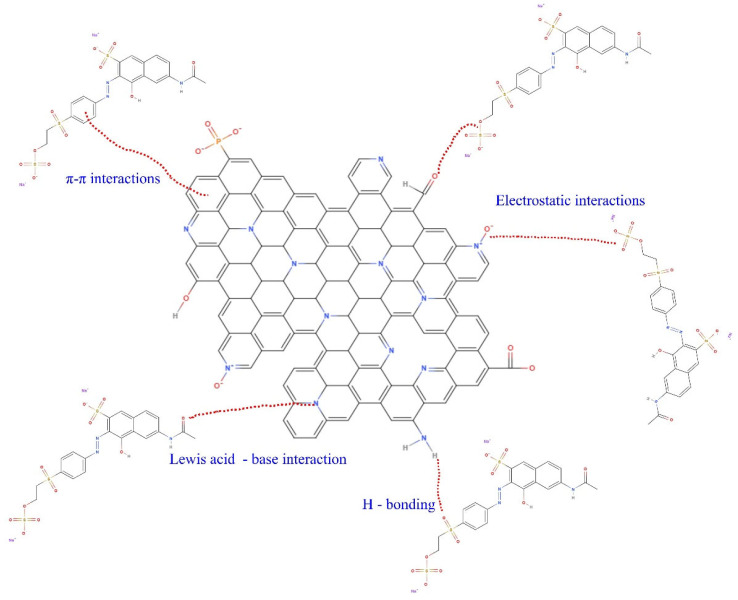
Mechanism of the adsorption of RO-16 onto SB-N-Biochar.

The mechanism of adsorption is graphically represented in Figure 8. Since the biochars have high specific surface areas, one of the dominant mechanisms of RO-16 adsorption is related to the pore-filling mechanism, achieved through the diffusion of the RO-16 molecules into the porous network of the biochars. In addition, the surface functionalities on the biochars’ surfaces play an important role in attracting and binding the RO-16 molecules [18,43,44]. The O-containing functional groups (hydroxyl and carboxyl groups) could act as an H-bonding donor to form hydrogen bonds with the RO-16 molecules [18,43,44]. Another mechanism acting on RO-16 removal can be seen in the Lewis acid-base interactions between N-doped biochar and RO-16; the N-doped biochar has N states (pyridinic-N, pyrrolic-N, and graphitic-N) and N functionalities that might donate electrons (N- and O) and act as Lewis bases [44]. Another important mechanism is seen in the electrostatic interactions. N doping creates positive clouds on the biochar carbon framework due to the electronegativity differences between carbon (2.55) and nitrogen (3.04), which enhance the electrostatic attraction of anionic dyes such as RO-16 [18]. During the doping process, nitrogen atoms withdraw the π-electrons from the biochar’s graphene layers, generating positive sites that act as acceptors of the π-electron and interact with the aromatic rings of the dye molecule (π-electron-enriched).

#### 3.2.6. Treatment of Synthetic Dye Effluents

SB-Biochar and SB-N-Biochar have proved their excellent ability to uptake a single molecule of RO-16; however, in this section, both adsorbents were tested to treat synthetic wastewater containing a mix of different dyes and inorganic/organic compounds that are commonly found in real wastewaters (see the composition of the effluents in Supplementary Table S1).

**Figure 9:**
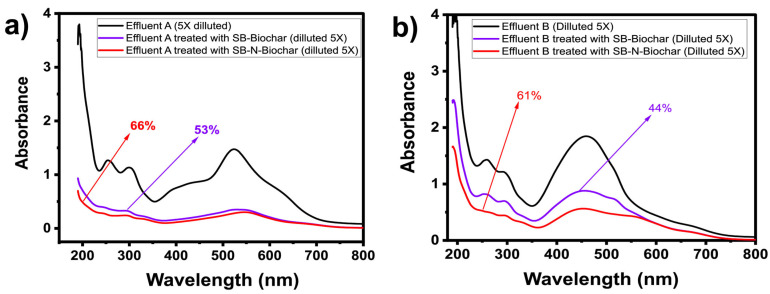
Adsorption of synthetic effluents. (**a**) Effluent A and (**b**) effluent B. See Supplementary Table S1 for the compositions and types of the effluents. Conditions for the effluent adsorption: 298 K and an adsorbent dosage of 1.5 g L^−1^, performed at 250 rpm.

For the proper calculation of the amounts of the compounds removed from the effluents, the UV-Vis absorption spectra before and after the adsorption process were recorded at wavelengths varying from 190 to 800 nm (see Figure 9). The overall contaminant removal percentage was calculated, using methods that have been described elsewhere [45,46,47], by integrating the area under the absorption spectrum.

For effluent A, the removal capacities were 53% and 66% for SB-Biochar and SB-N-Biochar, respectively. For effluent B, the removal capacities were 44% and 61% for SB-Biochar and SB-N-Biochar, respectively. As discussed previously, the SB-N-Biochar demonstrated better adsorptive performance compared to SB-Biochar, while for the effluent treatment, the doped sample also exhibited better adsorptive properties. Taking into account the fact that both synthetic effluents are complex, concentrated mixtures of dyes and other compounds, it can be suggested that both adsorbents could be successfully employed in the treatment of real-world colorful effluents.

#### 3.2.7. Regeneration Studies

The reusability of the adsorbent during multiple adsorption cycles is an important piece of information for evaluating the real-world adsorption applicability because it makes the adsorption process much more affordable, efficient, and environmentally friendly [48]. Aiming to understand the possible reusability/cyclability of the SB-N-Biochar, the samples underwent five consecutive adsorption–desorption tests. The RO-16 showed its highest adsorption capacity under acidic conditions; a basic eluent composed of 0.25 M of NaOH + 20% ethanol was employed in the desorption tests of RO-16 on biochars. The desorption tests were performed using the same procedure as in the adsorption tests. First, 50 mg of the RO-16 loaded biochar was put into contact with the eluent (0.25 M of NaOH + 20% ethanol) and was then stirred at 150 rpm for 2 h. The RO-16 concentration was quantified after each adsorption step via UV–vis spectrophotometry.

**Figure 10:**
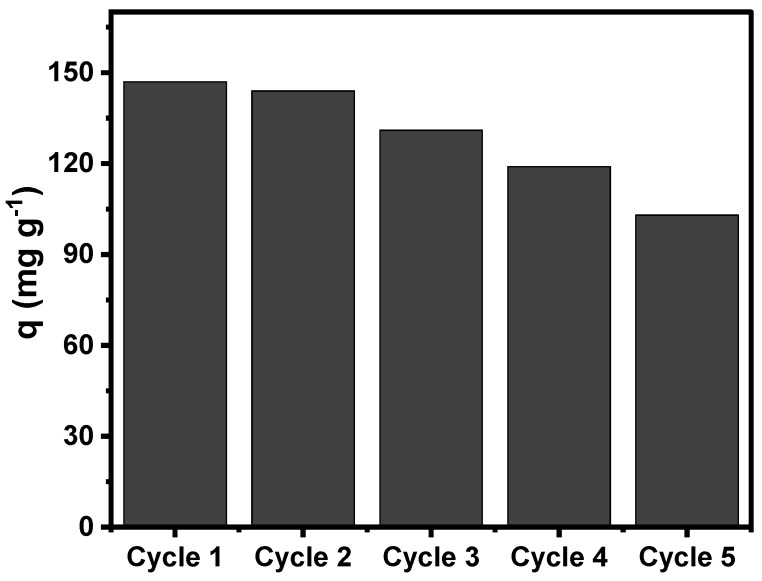
Cyclability tests of SB-N-Biochar over five cycles of RO-16 adsorption–desorption.

Figure 10 illustrates the desorption-adsorption cycles of RO-16 onto the SB-N-Biochar sample. It was observed that the adsorption capacities of the biochar suffered a small decrease, but they still maintained very good sorption efficiency after five cycles, with a reduction from 147 mg g^−1^ at the first cycle to 103 mg g^−1^ at the end of the fifth cycle. These results strongly indicate that the N-doping process yielded an adsorbent with satisfactory recyclability that could be used multiple times. This makes the process more sustainable and environmentally friendly.

## 4. Conclusions

In this study, spruce bark was used for the production of nitrogen-doped activated biochar. Melamine was used as the dopant and phosphoric acid was used as the chemical activator. A sample without the addition of melamine was used for comparison. Surface area analysis (BET), Raman spectrometry, and XPS were used for the characterization of the produced biochars. The capacity of the produced biochars to remove contaminants from water was determined using Reactive Orange 16 dye and synthetic effluents made up of several other dyes and inorganic compounds. Kinetic and isotherm studies were performed to characterize the produced biochars. The surface area (SSA) of the biochar was affected by the doping. The N-doped sample SSA was lower than that of the non-doped sample; however, the microporosity increased. XPS analysis showed that the doping process increased the number of N-surface groups on the surface of the biochar. The removal of RO-16 was higher at an acid pH, due to electrostatic interactions. The kinetics of adsorption measurements were described effectively with the Elovich model. The isotherms of adsorption were represented effectively by the Redlich–Peterson model, meaning that the adsorption process followed a hybrid mechanism and did not follow an ideal monolayer adsorption mechanism. The main mechanisms of adsorption regarding RO-16 and SB-N-Biochar were pore filling, hydrogen bonds, Lewis acid-base, and π-π between and electrostatic interactions. The adsorbent biochar materials were used to treat synthetic wastewater containing dyes and other compounds, and removal efficiencies of up to 66% were obtained. The regeneration tests have demonstrated that the nitrogen-doped biochar could be recycled and reused easily, maintaining very good adsorption performance even after five cycles.

## Figures and Tables

**Table 1:** Surface area of each biochar type.

	SB-Biochar	SB-N-Biochar
BET SSA (m^2^ g^−1^)	793	734
Micropore area (m^2^ g^−1^)	147	397
Micropore area (%)	19	54
Mesopore area (m^2^ g^−1^)	647	337
Mesopore area (%)	81	46
Total pore volume (cm^3^ g^−1^)	0.73	0.68
Average pore width (nm)	3.68	5.46

**Table 2:** XPS elemental analysis of SB-Biochar and SB-N-Biochar (at. ratio %).

		XPS				N 1s States	
	C 1s	O 1s	N 1s	P ^2^p	N1	N2	N3
Non-doped biochar	65.02	30.10	0.23	4.65		0.23	
N-doped-biochar	63.87	27.57	4.10	4.30	0.81	1.23	2.05

N1 = Pyridinic; N2 = Graphitic/Pyrrolic; N3 = Oxydized.

**Table 3:** Kinetic parameters for RO-16 adsorption.

Models	SB-Biochar	SB-N-Biochar
**Pseudo-first-order**		
q_1_ (mg g^−1^)	40.1	85.1
k_1_ (min^−1^)	0.0101	0.0915
R^2^_adj_	0.8973	0.897
SD (mg g^−1^)	4.11	8.83
**Pseudo-second-order**		
q_2_ (mg g^−1^)	42.8	91.2
k_2_ (g mg^−1^ min^−1^)	0.00391	0.00162
R^2^_adj_	0.949	0.963
SD (mg g^−1^)	2.89	5.29
**Elovich**		
α (mg/g min)	67.5	96.6
B (mg g^−1^)	0.0179	0.0795
R^2^_adj_	0.976	0.991
SD (mg g^−1^)	1.97	2.68

**Table 4:** Equilibrium data regarding RO-16 adsorption.

Models	SB-Biochar	SB-N-Biochar
**Langmuir**		
q_e_ (mg g^−1^)	113	152
k_1_ (min^−1^)	0.0056	0.134
R^2^_adj_	0.979	0.841
SD (mg g^−1^)	4.64	23.7
**Freundlich**		
k_F_ ((mg g^−1^)(mg L^−1^)^−1/nF^)	6.54	60.2
n_F_ (dimensionless)	2.50	6.51
R^2^_adj_	0.989	0.9825
SD (mg g^−1^)	3.30	7.50
**Redlich–Peterson**		
*K* _RP_ (L mg^−1^)	2.8 × 10^10^	1.8 × 10^10^
*A* _RP_	4.3 × 10^9^	3.0 × 10^11^
*β* _RP_	0.600	0.846
R^2^_adj_	0.999	0.999
SD (mg g^−1^)	1.91	9.044

**Table 5:** Comparison of the q_max_ values for Reactive Orange 16 dye using different adsorbents.

Adsorbents	q_max_ (mg g^−1^)	T (°C)	pH	Ref.
Mesoporous carbon from fish scales	114.2	50	7.0	[33]
Chitosan/sepiolite composite	190.9	30	3.0	[34]
Coffee waste and red mud composite	144.8	50	2.0	[35]
Chitosan-TPP/TiO_2_ nanocomposite	618.7	40	4.0	[36]
Paper sludge activated carbon	178.0	30	2.0	[37]
*Phyllanthus reticulatus* activated carbon	15	30	2.0	[38]
Chitosan-glyoxal/fly ash/Fe_3_O Composite	112.5	40	4.0	[39]
Brewery yeast activated carbon	340	35	3.0	[40]
Landfill sludge biosorbent	159	25	4.0	[41]
Magnetic chitosan-polyvinyl alcohol blend (fly ash modified)	123.8	30	4.0	[42]
SB-Biochar	100.6	25	6.0	This work
SB-N-Biochar	173.9	25	6.0	This work

## Data Availability

Data can be made available upon request.

## Supplementary Materials

The following supporting information can be downloaded at: https://www.mdpi.com/article/10.3390/nano13142045/s1, “Information about Adsorption on Kinetic and Equilibrium Models and Calculations”; Table S1: Chemical composition of simulated synthetic effluents; Figure S1: (A) Structural formulae of reactive orange 16. (B) Optimized three-dimensional structural formulae of RO-16.
