# Correction: Ekman et al. Synthesis, Characterization, and Adsorption Properties of Nitrogen-Doped Nanoporous Biochar: Efficient Removal of Reactive Orange 16 Dye and Colorful Effluents. *Nanomaterials* 2023, *13*, 2045

**DOI:** 10.3390/nano16120706

**Published:** 2026-06-09

**Authors:** Simon Ekman, Glaydson Simoes dos Reis, Ewen Laisné, Julie Thivet, Alejandro Grimm, Eder Claudio Lima, Mu. Naushad, Guilherme Luiz Dotto

**Affiliations:** 1 Umeå University, SE-901 83 Umeå, Sweden; simonoscekman@gmail.com; 2 Department of Forest Biomaterials and Technology, Biomass Technology Centre, Swedish University of Agricultural Sciences, SE-901 83 Umeå, Sweden; ewen.laisne@mines-albi.fr (E.L.); julie.thivet@enscm.fr (J.T.); alejandro.grimm@slu.se (A.G.); 3 IMT Mines Albi-Carmaux, 81000 Albi, France; 4 Ecole Nationale Supérieure de Chimie de Montpellier, 34090 Montpellier, France; 5 Federal University of Rio Grand do Sul (UFRGS), Porto Alegre 90010-150, RS, Brazil; profederlima@gmail.com; 6 Department of Chemistry, College of Science, King Saud University, P.O. Box 2455, Riyadh 11451, Saudi Arabia; shad123@gmail.com; 7 Research Group on Adsorptive and Catalytic Process Engineering (ENGEPAC), Federal University of Santa Maria, Av. Roraima, 1000-7, Santa Maria 97105-900, RS, Brazil; guilherme_dotto@yahoo.com.br

Error in Figure/Table

In the original publication [1], there was an error in Figure 3. The figure was incorrect as published and has been replaced with the correct one. The XPS analysis results were presented without the raw spectra; additionally, the deconvolution was not performed accurately. The error has now been corrected, and the updated Figure 3 and Table 2 are shown below.

Text Correction

A correction has been made in Subsection *3.1. Physicochemical Characterization of the Produced Biochars*, Paragraphs 5 and 6. The new text is as follows:

Figure 3c,d show the high-resolution deconvoluted N 1 s spectrum for SB-Biochar and SB-N-Biochar, respectively. Only one peak at 399.6 eV was observed in SB-Biochar, corresponding to graphitic/pyrrolic-N. However, SB-N-Biochar (see Figure 3d) exhibits three separate peaks at 398.3 eV, 400.4 eV, and 403.5 eV, corresponding to pyridinic-N (N1), graphitic/pyrrolic-N (N2), and oxidized-N (N3), respectively [23]. XPS analysis has shown that nitrogen atoms were successfully incorporated into the carbonaceous matrix. Moreover, phosphorus was detected in both materials, which is due to its activation with H_3_PO_4_.

Through XPS analysis, it was possible to obtain a quantitative analysis of each element (see Table 2). The nitrogen content in SB-Biochar was only 0.2%, while that in SB-N-Biochar was 4.1%, which confirms that the doping process incorporated large amounts of N into the biochar structure. Nitrogen doping produced an adsorbent (SB-N-Biochar) with higher amounts of N, indicating that its surface contains larger amounts of functional groups, which directly correlate with the better adsorptive properties gained through electrostatic interactions between these functionalities and the adsorbates.

The authors state that the scientific conclusions are unaffected. This correction was approved by the Academic Editor. The original publication has also been updated.

## Figures and Tables

**Figure 3:**
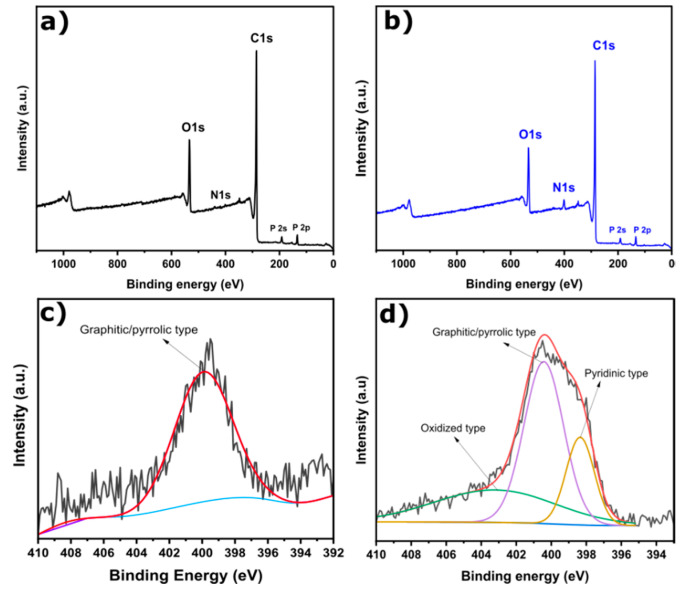
X-ray photoelectron spectroscopy (XPS) analysis of the SB-Biochar and SB-N-Biochar: (**a**) Survey spectrum of SB-Biochar; (**b**) survey spectrum of SB-N-Biochar; (**c**) deconvoluted N 1 s peaks for SB-Biochar; (**d**) deconvoluted N 1 s peaks for SB-N-Biochar.

**Table 2:** XPS elemental analysis of SB-Biochar and SB-N-Biochar (at. ratio %).

		XPS				N 1s States	
	C 1s	O 1s	N 1s	P ^2^p	N1	N2	N3
Non-doped biochar	65.02	30.10	0.23	4.65		0.23	
N-doped-biochar	63.87	27.57	4.10	4.30	0.81	1.23	2.05

N1 = Pyridinic; N2 = Graphitic/Pyrrolic; N3 = Oxydized.
