# Supplementary material for: Synthesis, Characterization, and Adsorption Properties of Nitrogen-Doped Nanoporous Biochar: Efficient Removal of Reactive Orange 16 Dye and Colorful Effluents
Source: Nanomaterials (Basel). 2023 Jul 11;13(14):2045. doi: 10.3390/nano13142045 (PMC10385902; doi:10.3390/nano13142045)
Supplement: Supplementary file 1 [file nanomaterials-13-02045-s001.zip › nanomaterials-2474923-supplementary.pdf]

nanomaterials

Supplementary Materials

Synthesis, Characterization, and Adsorption Properties of Nitrogen-Doped Nanoporous Biochar: Efficient Removal of Reactive Orange 16 Dye and Colorful Effluents

Simon Ekman [1,2], Glaydson Simoes dos Reis [2,*], Ewen Laisné [2,3], Julie Thivet [2,4], Alejandro Grimm [2], Eder Claudio Lima [5], Mu. Naushad [6] and Guilherme Luiz Dotto [7]

[1] Umeå University, SE-901 83 Umeå, Sweden
[2] Department of Forest Biomaterials and Technology, Biomass Technology Centre, Swedish University of Agricultural Sciences, SE-901 83 Umeå, Sweden
[3] IMT Mines Albi-Carmaux, 81000 Albi, France
[4] Ecole Nationale Supérieure de Chimie de Montpellier, 34090 Montpellier, France
[5] Federal University of Rio Grand do Sul (UFRGS), 90010-150 Porto Alegre, RS, Brazil
[6] Department of Chemistry, College of Science, King Saud University, P.O. Box 2455, Riyadh 11451, Saudi Arabia
[7] Research Group on Adsorptive and Catalytic Process Engineering (ENGEPAC), Federal University of Santa Maria, Av. Roraima, 1000-7, Santa Maria 97105-900, RS, Brazil
* Correspondence: glaydson.simoes.dos.reis@slu.se

2.3. Adsorption experiments

De-ionized water was used for the preparation of the RO-16 dye solutions. The batch mode was employed to evaluate the applicability of the non-doped and nitrogen doped biochars in removing RO-16 dye from aqueous solutions. The effect of the pH and initial dye concentration on RO-16 dye adsorption were investigated. All adsosption experiments were performed using 30 mg of each biochar into 20 mL of RO-16 dye using 50.0 mL Falcon tubes. For isotherms studies, RO-16 initial solution concentrations from 70 to 1200 mg L^{-1} were employed in the experiments. The effect of the pH was determined by varying the pH from 2 to 10. The kinetic RO-16 dye removal on biochars was studied varying contact time from 0 to 300 min with an RO-16 dye initial concentration of 200 mg L^{-1}. All adsorption experiments were performed at a constant shaking speed of 200 rpm. After adsorption, the residual solutions of RO-16 were quantified using a UV-Visible spectrophotometer (Shimadzu 1800) at a maximum wavelength of 494 nm. The removal capacity and the percentage of RO-16 removal are obtained from Equations 1 and 2, respectively.

$$q=(C_0-C_f)\frac{V}{m} \quad (1)$$

Copyright: © 2023 by the authors. Licensee MDPI, Basel, Switzerland. This article is an open access article distributed under the terms and conditions of the Creative Commons Attribution (CC BY) license (https://creativecommons.org/licenses/by/4.0/).

$$R=\frac{(C_0-C_f)}{C_0}100 \quad (2)$$

q is the quantity of RO-16 removed (mg g^{-1}). C_o and C_f are the initial and final RO-16 concentrations (mg L^{-1}), respectively. m is the mass of biochars (g), and V is the used volume of RO-16 in the adsorption experiments (L).

2.4. Kinetic, Equilibrium models, and synthetic effluents

Pseudo-first-order: $q_t = q_e\,(1-\exp^{-k_1.t})$ (3)

pseudo-second-order: $q_t = \frac{k_2.q_e^2.t}{1+q_e.k_2.t}$ (4)

Elovich: $q_t = \frac{1}{\beta}\ln(1+\propto \beta t)$ (5)

where, ***t*** denotes the contact time (min); q_t, q_e are the adsorption capacities at time t and at equilibrium, respectively (mg/g); k_1 is the pseudo-first-order rate constant (L/min); k_2 is the pseudo-second-order rate constant (g/mg min); k_N is the general-order constant rate [$(g/mg)^{n-1}$)/min], and **n** is the dimensionless general-order adsorption rate; α is the initial sorption rate (mg $g^{-1}min^{-1}$), and β is the desorption constant (g.mg^{-1}) during any one experiment.

Equilibrium of adsorption isotherms

Langmuir: $q_e = \frac{q_{max}.K_L.C_e}{1+K_L.C_e}$ (6)

Freundlich: $q_e = K_F.C_e^{1/n_F}$ (7)

Redlich-Peterson: $q_e = \frac{K_{RP}\,.\,C_e}{1+a_{RP}\,.C_e{}^{\beta}}$ (8)

where, $\mathbf{q_e}$ denotes the amount of adsorbate adsorbed at the equilibrium (mg/g); $\boldsymbol{C_e}$ is the adsorbate concentration at equilibrium (mg/L); $\boldsymbol{q_{max}}$ is the maximum adsorption capacity of the adsorbent (mg/g); $\mathbf{K_L}$ and $\mathbf{K_s}$ are the Langmuir and Sips equilibrium constant (L/mg), respectively; $\mathbf{K_F}$ is the Freundlich equilibrium constant [(mg/g) $(mg/L)^{-1/n_F}$]; $\mathbf{n_F}$ is the dimensionless exponents of the Freundlich; a_{RP} is the affinity constant of Redlich–Peterson related to the equilibrium constant (L/ mg)β. K_{RP} (L/g) is a constant that contains the maximum sorption capacity and the affinity of adsorbent-adsorbate. β is the exponent of Redlich–Peterson isotherm that is dimensionless. By definition $0 < \beta \leq 1$.

The quality control of adsorption data is further described elsewhere [26-32]. Nonlinear fitting of kinetic and equilibrium data was performed using the Microcal Origin 2020 software. The nonlinear fitting was obtained using the Simplex method and the Levenberg–Marquardt algorithm for performing this task. The adequacy of the kinetic and equilibrium models was statistically assessed employing the adjusted determination coefficient (R^2_{adj}) and the standard deviation of residues (SD) [24-32] shown in equations 9 and 10 below.

$$R^2_{adj} = 1 - (1 - R^2).\left(\frac{n-1}{n-p-1}\right) \quad (9)$$

$$SD = \sqrt{\left(\frac{1}{n-p}\right).\sum_{i}^{n}\left(q_{i,exp} - q_{i,model}\right)^2} \quad (10)$$

where $q_{i,\,model}$ is the individual model sorption capacity expected by the model; $q_{i,\,exp}$ is the individual experimentally measured *sorption capacity*; $\overline{q}_{i,exp}$ is the average of all measured experimental *sorption capacities*; n is the number of experiments performed; p is the number of model parameters in the fitting model.

The R^2_{adj} and SD values were used to compare different kinetics and equilibrium models. The best-fitted model would present the R^2_{adj} closer to 1.00 and the lowest SD values [24-32].

Supplementary Table S1 Effluent compositions and concentrations

Compounds	Concentration (mg L^{-1})		λ_{max} (nm)
Effluent	A	B	
Acid red 18	50	50	507
Reactive orange 16	50	50	494

Reactive blue 4	50	50	595
Methylene Blue	50	50	668
Evans blue	50	50	468
Phenol Red	50	50	550
Crystal Violet	50	-	590
Methyl Red	50	-	507
Bismarck Brown	-	50	468
Methyl Orange	-	50	522
Sodium Dodecyl	25	25	-
Sodium sulfate	25	25	-
Ammonium chloride	20	25	-
Sodium acetate	20	25	-
pH	5.4	5.2	-

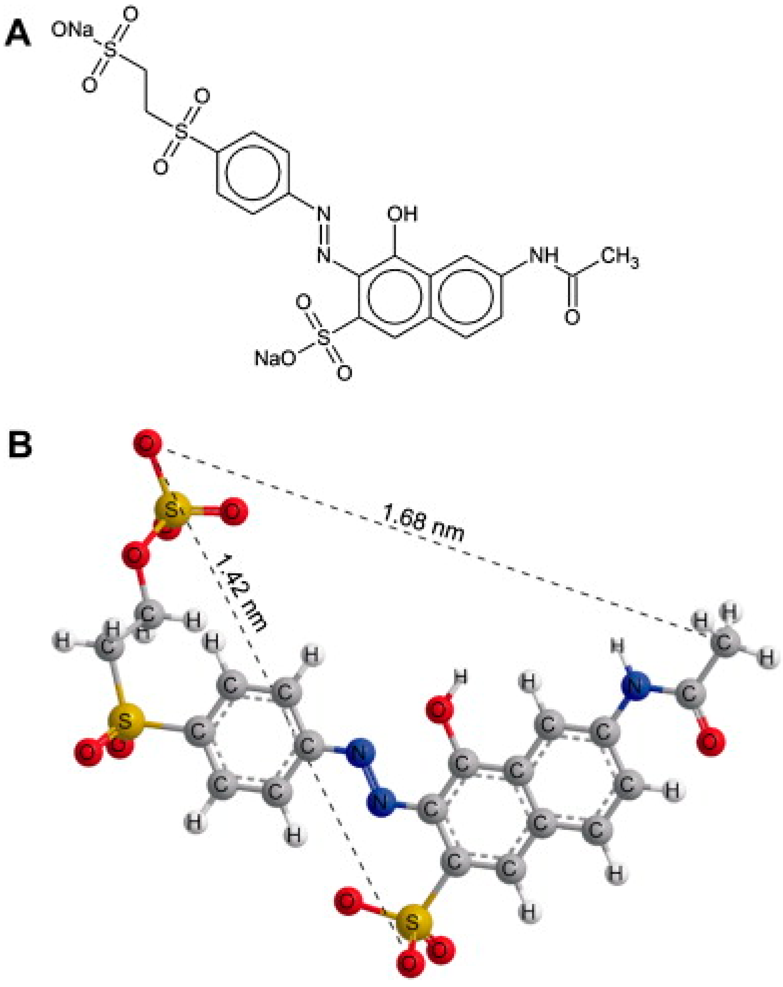

Figure S1. (A) Structural formulae of reactive orange 16. (B) Optimized three-dimensional structural formulae of RO-16. The dimensions of the chemical molecule were calculated using ChemBio 3D Ultra version 11.0.
